# Second International Consensus Conference on lesions of uncertain malignant potential in the breast (B3 lesions)

**DOI:** 10.1007/s10549-018-05071-1

**Published:** 2018-11-30

**Authors:** Christoph J. Rageth, Elizabeth A. M. O’Flynn, Katja Pinker, Rahel A. Kubik-Huch, Alexander Mundinger, Thomas Decker, Christoph Tausch, Florian Dammann, Pascal A. Baltzer, Eva Maria Fallenberg, Maria P. Foschini, Sophie Dellas, Michael Knauer, Caroline Malhaire, Martin Sonnenschein, Andreas Boos, Elisabeth Morris, Zsuzsanna Varga

**Affiliations:** 1 Département de Gynécologie et d’Obstétrique, Centre du sein, Hôpitaux Universitaires de Genève, Bd de la Cluse 30, 1211 Geneva 14, Switzerland; 2 The Rose Centre, St George’s University Hospitals NHS Foundation Trust, Perimeter Road, London, SW17 0QT UK; 3 Breast Imaging Service, Department of Radiology, Memorial Sloan Kettering Cancer Center, 300 E 66th St, New York, NY 10065 USA; 4 Department of Medical Services, Institute of Radiology, Kantonsspital Baden, im Ergel, 5404 Baden, Switzerland; 5 Zentrum Radiologie der Niels-Stensen-Kliniken; Marienhospital Osnabrück, Bischofsstraße 1, 49074 Osnabrück, Germany; 6 Institut für Pathologie am Dietrich-Bonhoeffer-Klinikum, Salvador-Allende-Straße 30, 17036 Neubrandenburg, Germany; 7 Brust-Zentrum Zürich, Seefeldstr. 214, 8008 Zurich, Switzerland; 8 Interventional and Pediatric Radiology, Department of Diagnostic, Inselspital, University Hospital Bern, Freiburgstrasse 10, 3010 Bern, Switzerland; 9 Department of Biomedical Imaging and Image-guided Therapy, Allgemeines Krankenhaus, Medical University of Vienna, Währinger Gürtel 18-20, 1090 Vienna, Austria; 10 Department of Radiology, University Hospital, Ludwig Maximilian University Munich, Marchioninistr. 15, 81377 Munich, Germany; 11 Department of Biomedical and Neuromotor Sciences, Unit of Anatomic Pathology at Bellaria Hospital, University of Bologna, Via Altura 3, 40139 Bologna, Italy; 12 Clinic of Radiology and Nuclear Medicine, University Hospital Basel, University of Basel, Petersgraben 4, 4031 Basel, Switzerland; 13 Breast Center St. Gallen, Cantonal Hospital St. Gallen, Rorschacher Str. 95, 9007 St. Gallen, Switzerland; 14 Imaging Department, Institut Curie, PSL Research University, Paris, France; 15 Division of Radiology, Breast Center Bern (Brustzentrum Bern), Klinik Engeried, Lindenhofgruppe AG, Riedweg 15, 3012 Bern, Switzerland; 16 Institute of Diagnostic and Interventional Radiology, University Hospital Zurich, Rämistr. 100, 8091 Zurich, Switzerland; 17 Institute of Pathology and Molecular Pathology, University Hospital Zurich, Switzerland Schmelzbergstrasse 12., 8091 Zurich, Switzerland; 18 Ringlikerstrasse 53, 8142 Uitikon Waldegg, Switzerland

**Keywords:** B3 lesions, Vacuum-assisted biopsy, Consensus, Breast, Uncertain malignant potential, Breast surgery

## Abstract

**Purpose:**

The second International Consensus Conference on B3 lesions was held in Zurich, Switzerland, in March 2018, organized by the International Breast Ultrasound School to re-evaluate the consensus recommendations.

**Methods:**

This study (1) evaluated how management recommendations of the first Zurich Consensus Conference of 2016 on B3 lesions had influenced daily practice and (2) reviewed current literature towards recommendations to biopsy.

**Results:**

In 2018, the consensus recommendations for management of B3 lesions remained almost unchanged: For flat epithelial atypia (FEA), classical lobular neoplasia (LN), papillary lesions (PL) and radial scars (RS) diagnosed on core-needle biopsy (CNB) or vacuum-assisted biopsy (VAB), excision by VAB in preference to open surgery, and for atypical ductal hyperplasia (ADH) and phyllodes tumors (PT) diagnosed at VAB or CNB, first-line open surgical excision (OE) with follow-up surveillance imaging for 5 years. Analyzing the Database of the Swiss Minimally Invasive Breast Biopsies (MIBB) with more than 30,000 procedures recorded, there was a significant increase in recommending more frequent surveillance of LN [65% in 2018 vs. 51% in 2016 (*p* = 0.004)], FEA (72% in 2018 vs. 62% in 2016 (*p* = 0.005)), and PL [(76% in 2018 vs. 70% in 2016 (*p* = 0.04)] diagnosed on VAB. A trend to more frequent surveillance was also noted also for RS [77% in 2018 vs. 67% in 2016 (*p* = 0.07)].

**Conclusions:**

Minimally invasive management of B3 lesions (except ADH and PT) with VAB continues to be appropriate as an alternative to first-line OE in most cases, but with more frequent surveillance, especially for LN.

## Introduction

Lesions of uncertain malignant potential in the breast (B3 lesions) represent a heterogeneous group of abnormalities with an overall risk for malignancy of 9.9%–35.1% after total resection [1]. Historically open surgical excision has been recommended for all B3 lesions; however, over the last decade there has been a trend towards minimally invasive breast biopsy or percutaneous excision using a vacuum-assisted device where larger volumes of tissue can be removed compared to core biopsy, equivalent to a small-wide local excision while retaining the same diagnostic accuracy as open surgery [2], but with the obvious benefits of saving the patient a surgical procedure, and cost. Underestimates of malignancy in excised B3 lesions range up to 35% and are associated primarily with increasing size of the lesion and the presence of atypia rather than the nature of the mammographic abnormality (e.g., calcification vs. mass or architectural distortion) [3]. Several studies also indicate that B3 lesions are predominantly upgraded to ductal carcinoma in situ (DCIS) and low-grade invasive tumors [1, 3–6].

The evidence base for the outcome and behavior of B3 lesions in the literature is accruing. Management and practice vary greatly from country to country, although there is a trend universally for more conservative management as an alternative to open surgery. The 2016 recommendations from the first International Consensus Conference on B3 lesions [7] during the biannual International Breast Ultrasound School (IBUS) course were well accepted by many breast units in different countries. The purpose of the second International Consensus Conference in 2018 was to re-evaluate how recommendations for the management and follow-up surveillance of B3 lesions in the breast had influenced daily practice, review the most recent literature, and investigate the trend towards less open surgery and appropriate surveillance.

### Methodology

The second International Consensus Conference on lesions of uncertain malignant potential (B3) was held with international experts as part of the IBUS seminar in March 2018. The meeting in March 2018 had 70 participants with an additional 19 multidisciplinary expert panel members (including all the aforementioned authors) comprising 55% radiologists, and 45% other (including pathologists, surgeons, and gynecologists) with 68% having more than 10 years’ experience in breast imaging. All participants were invited to vote on all recommendations and between 60 and 80 (depending on the question asked) decided to vote.

A new analysis of the Swiss Minimally Invasive Breast Biopsy group (MIBB) Database was performed and presented (histology from 31,574 VABs). The Swiss MIBB group—a subgroup of the Swiss Society of Senology founded in 2007—has collected data for 11 years on each diagnostic or therapeutic VAB performed in Switzerland. To evaluate the impact of the B3 guidelines from the first International Consensus Conference in the management and surveillance of B3 lesions, the data were compared between 2007 and 2015 and 2016–2017 using the Chi-squared test.

Recommendations for management of B3 breast lesions following histological diagnosis were either (i) surveillance (defined as 6 monthly or yearly mammography and/or ultrasound, depending on their imaging findings), (ii) VAB excision, or (iii) open excision.

Following presentations of each B3 lesion in detail with an update of the published literature since the first International Consensus Conference, three questions were asked in turn regarding each of the six B3 lesions [8]:

1. If a core-needle biopsy (CNB) returned a B3 lesion on histology, should the lesion be excised?
2. If so, should it be excised using vacuum-assisted biopsy (VAB) or open surgical excision (OE)?
3. If the VAB returned a B3 lesion on histology and if the lesion was completely removed on imaging, is surveillance acceptable or should a repeat VAB or OE be performed?

A panel discussion followed the voting and consensus recommendations were agreed for the management of each B3 lesion along with decisions on surveillance.

## Results

### Analysis of the MIBB database

From 2007 until 2017, a total of 31,574 VABs were entered in the database. 6,020 cases (19.1%) showed a B3 lesion (4339 were pure and the other ones were combined B3 lesions).

Table 1 shows the pure B3 lesions together with the final histology in those which had a subsequent open surgery and upgrade rates.

**Table 1:** Pure B3 lesions together with the final histology in the cases, which had a subsequent open surgical excision (OE)

Pure B3 histology	*N*	With subsequent OE	Total upgrade	Upgrade to DCIS OR pleomorphic LN	Upgrade to IC	No upgrade
ADH	943	591 (62.7%)	149 (25.2%)	119 (20.1%)	30 (5.1%)	408 (69.0%)
FEA	994	249 (25.1%)	40 (16.1%)	22 (8.8%)	18 (7.2%)	181 (72.7%)
LN	701	268 (38.2%)	68 (25.4%)	35 (13.1%)	33 (12.3%)	178 (66.4%)
PL	1251	272 (21.7%)	21 (7.7%)	16 (5.9%)	5 (1.8%)	217 (79.8%)
PT	35	4 (11.4%)	0	0	0	4 (100%)
RS	415	75 (18.1%)	6 (8%)	5 (6.7%)	1 (1.3%)	60 (80.0%)

*IC* invasive cancer

Table 2 shows recommendations made to the patients following VAB. Between 2016 and 2017, surveillance was recommended more frequently for all B3 lesions following VAB, but this was only significant for the following lesions: FEA (72% vs. 61.5%: *p* = 0.005), LN (64.9% vs. 51%; *p* = 0.004), and PLs (76% vs. 69.7%; *p* = 0.04).

**Table 2:** Pure B3 lesions with the recommendations after the VAB comparing two time periods 2016–2017 versus 2007–2015

Pure B3 histology	N MIBBs		OE Recommended		Surveillance recommended		Recommendation of surveillance difference between 2 time periods in %
	2007–2015	2016–2017	2007–2015	2016–2017	2007–2015	2016–2017	
ADH	779	160	549 (70.5%)	113 (70.6%)	181 (23.2%)	41 (25.6%)	2.4 (*p* = 0.52)
FEA	786	207	247 (31.4%)	52 (25.1%)	483 (61.5%)	149 (72%)	10.5* (*p* = 0.005)
LN	561	131	236 (42.1%)	42 (32.1%)	286 (51%)	85 (64.9%)	13.9* (*p* = 0.004)
PL	961	288	217 (22.6%)	57 (19.8%)	670 (69.7%)	219 (76%)	6.3* (*p* = 0.04)
PT	22	13	8 (36%)	3 (23%)	14 (64%)	9 (69%)	5.6 (*p* = 0.74)
RS	316	99	80 (25.3%)	18 (18.2%)	212 (67.1%)	76 (76.8%)	9.7 (*p* = 0.07)

*Significant result

*OE* Open surgical excision

### General recommendations of the panel members of the consensus conference

#### Acceptable rates for the risk of underestimation

In 2016, the panel of the first International Consensus Conference on B3 lesions stated that every B3 lesion should be discussed at a multidisciplinary meeting (MDM). If an MDM makes the decision not to perform open surgery after a diagnosis of a B3 lesion following VAB, it means balancing risks (e.g., having to undergo a surgery under anesthesia which produces a scar) and benefits (e.g., not risking underestimating a lesion, which could be or develop towards an invasive cancer). Therefore in 2018, the question asked was: What is an acceptable underestimation rate for DCIS or IC?

69 participants gave answers for upgrade to IC: < 2.5%: 36 (53%); < 5%: 23 (34%); < 7.5%: 8 (12%); and < 10%: 2 (3%).

68 participants gave answers for upgrade to DCIS: < 5%: 15 (22%); < 10%: 40 (59%); < 15%: 9 (13%); and < 20%: 4 (6%). Therefore, overall underestimation rates for the majority of the panel members were that it should not exceed 5% for IC and 10% for DCIS.

#### Reasons for recommending an open biopsy instead of surveillance

The panel also discussed which circumstances would argue for performing an open biopsy instead of surveillance only. Discrepancy between histology and imaging was by far the most important factor. For example, if a solid lesion and not only microcalcifications are seen, then histology should correspond to this finding. Further strong arguments for performing a subsequent open biopsy or a repeat VAB were a residual lesion and lesion size. The larger a lesion is, the more likely an open biopsy should be recommended. For an ultrasound-guided VAB, the size should usually not exceed 2.5 cm. Elevated personal risk, the presence of a solid lesion on ultrasound, associated calcifications within the lesion, and absence of calcifications within the lesion were also considered.

### Recent literature

Recent manuscripts dealing with B3 lesions were selected for presentation and discussion at the conference. Many of the papers document upgrade rates in following open excision and the risk of developing a cancer during the years following a diagnosis of a B3 lesion. In some of the manuscripts, CNB and VAB were not well differentiated. CNB, often also called microbiopsy, should be used for CNB performed with devices smaller or equal to 14G. The term VAB, often called macrobiopsy, would therefore be reserved for larger needle devices (typically 7 to 11G). Since upgrade rates depend on the amount of tissue, which is available for the pathologist for examination, this distinction is important.

### Atypical ductal hyperplasia (ADH)

#### Histological criteria of ADH

ADH is a low-grade neoplastic intraductal proliferation. The histological criteria of ADH include quantitative features of low-grade atypia as monomorphic nuclei with clear membranous borders and secondary intraluminal adenoid architecture. As quantitative features, restriction to one terminal ductal-lobular unit (TDLU) is usually ≤ 2 mm in maximal extension, whereas the histological as immunophenotypical features of an ADH lesion are the same as at low-grade DCIS. Intraductal ADH cell proliferations are negative for high molecular weight cytokeratins and strongly and diffusely positive for estrogen receptors in the same pattern as seem at low-grade DCIS. The differential diagnosis between ADH and DCIS is based on size only. Therefore, a low-grade in situ neoplastic lesion with qualitative features of ADH cannot definitely be separated from a part of a larger low-grade DCIS based on findings in minimal invasive breast biopsy (CNB or VAB) alone. The European Working Group on Breast Screening Pathology recommends that it should always be kept in mind that such proliferations at a biopsy may represent the periphery of a more established lesion of DCIS [9].

### Underestimation risk associated with ADH at VAB

The dilemma in decision making on management of an ADH-like lesion at MIBB is the uncertainty whether it represents a part of a larger DCIS or is an isolated lesion. There is only limited information on histological, imaging, and clinical factors, which can reliably predict the answer. These include lesion size and number of ADH foci in biopsy specimens, radiological features, needle type, and association with calcification and individual cell necrosis. Until now, none of these features can reliably exclude an upgrade in the surgical specimen. However, risk factors for underestimation of malignancy include multifocality with more than 2 foci of ADH on CNB, and associated individual cell necrosis, this latter might be suggestive but definitely not affirmatively diagnostic of a low-grade DCIS. In addition, lack of radiological-pathological correlation as lack of calcification in MIBB specimens on VAB performed for mammographically suspicious calcifications as well as ADH-like lesions as only histopathological finding in biopsies taken for mass lesions on imaging. Conflicting results of several studies analyzing the risk factors of synchronous malignancy in MIBB with ADH published in recent years as the large range of their underestimation rates (2%–50%), as summarized in Table 3, seems to be depending on the type of biopsy performed (CNB or VAB), age (> 50 years), and on associated microcalcification on imaging. But above all, upgrade rates are generally higher in biopsies without any pathological correlation to the target lesion in imaging. Table 3 summarizes the literature update on ADH since 2015.

**Table 3:** Summary of the recent literature on ADH since 2015

Author and year	Number of patients analyzed or type of publication if no patients have been analyzed (e.g., review or comment)	Findings	Conclusions
Ahn et al. 2016 [10]	*n* = 103 Upgrade	Underestimation rates FEA (5.9%) FEA + ADH (44.4%) ADH 27.3%	Recommend OE especially if calcification is present
Badan et al. 2016 [11]	*n* = 40 Upgrade	Underestimation rate ADH in CNB (50%) ADH in VAB (25%)	Recommend OE
Co et al. 2018 [12]	*n* = 104	ADH in CNB (41%)	Suspicious mammogram correlates with upgrade
Collins et al. 2016 [13]	Association between extent of ADH/LN and BC risk	1–2 foci ADH (OR 3.5) 1–2 foci LN (OR 5.2) ≥ 3 foci ADH (OR 2.7) ≥ 3 foci LN (OR 8.0)	No influence of extent of ADH or LN on BC risk
Degnim et al. 2016 [14]	Association between extent of ADH /LN and BC risk	1–2 foci ADH (RR:2.65) 2 foci ADH (RR: 5.19) ≥ 3 foci ADH (RR 8.94) 1–2 foci LN (RR:2.58) 2 foci LN (RR: 3.49) ≥ 3 foci LN (RR 4.97)	BC risk increases with ADH/LN extension *p* < 0.001
Donaldson et al. 2018 [15]	*n* = 393 Upgrade	ADH/LN on CNB	No upgrade
Khoury et al. 2016 [16]	*n* = 100 Upgrade	Underestimation rate ADH in VAB (15%)	Extension and nb of positive cores correlate with upgrade
Latronico et al. 2018 [17]	Upgrade (*n* = 45) and long-term follow-up (*n* = 12)	Upgrade after ADH 45% BC (8%)	Recommend OE
Menen et al. 2017 [18]	*n* = 175 Follow-up after/wo surgery	BC 12% (after surgery) BC 5.6% (only follow-up) Contralateral BC only after surgery	Prior history of breast cancer was the only variable associated with subsequent breast cancer events (hazard ratio 12.53)
Menes et al. 2017 [19]	BC risk after ADH in CNB (*n* = 1727) OE (*n* = 635)	10-year cumulative BC risk 2.6% (CNB) 5.7% (OE)	BC risk after ADH diagnosis is higher
Mesurolle et al. 2014 [20]	*n* = 50 Upgrade ADH in CNB	Underestimation rate ADH in CNB (56%)	OE recommended
Pena et al. 2017 [21]	*n* = 399 Low BC risk after ADH in CNB	Underestimation rate ADH in CNB (16%) Low BC risk ADH in CNB (4–9%)	Low BC risk if (1) lack of necrosis and (2) 1–2 foci or ≥ 3 foci with ≥ 90% removal
Renshaw and Gould, 2016 [4]	Upgrade and Long-term clinical follow-up 175 ADH on CNB	Underestimation rate ADH in CNB (30.3%) BC after surgery (11.5%)	Immediate BC risk is higher for ADH than LN Long-term BC risk is higher for LN than ADH
Yu et al. 2015 [22]	Upgrade ADH in CNB (83)	Underestimation rate ADH in CNB 9.5%	Age, associated mass, and calcification distribution are independent factors for upgrade
Rageth et al. (data presented at the conference, but not yet published)	Upgrade and histological criteria 207 ADH cases (56 CNBs and 151 VABs)	Underestimation rate ADH in CNB 57% ADH in VAB 33%	Factors in upgrade (1) Method (CNB vs. VAB) (2) The presence of multifocality (3) Absence of associated calcification

Since upgrade rates in so-called lower-risk subgroups exceed the defined acceptable limits for underestimation (10% for DCIS and 5% for IC), OE is recommended in general even if the lesion seems to be completely excised by VAB. Surveillance instead of OE might be appropriate in special situations (especially in older age) since most of the IC that develop after ADH are small low-grade cancers. Surveillance is also necessary after OE because such patients are at a higher risk of developing cancer also distant from the excised ADH lesion and also in the contralateral breast.

### Voting

If a CNB returned ADH on histology,

100% of the participants thought the lesion should be excised. 21% thought therapeutic VAB excision was acceptable and 74% thought therapeutic open surgical excision should be performed. 5% were undecided.

If a VAB returned ADH on histology,

51% of the participants thought that therapeutic open surgical excision should be performed and 42% thought that surveillance was adequate (Table 9).

### Consensus recommendation of the panel

A lesion containing ADH diagnosed by CNB or VAB should undergo open surgical excision. Surveillance can be justified only in special situations after discussion at the MDM (Table 10).

### Flat epithelial atypia (FEA)

#### Histological criteria of FEA

FEA is a low-grade neoplastic lesion consisting of a few layers of neoplastic columnar type cells with low-grade (monomorphic) atypia without any secondary architecture (flat architecture). The immunophenotype of a FEA lesion is identical to that of a low-grade DCIS, which is negative for basal cytokeratins and positive for estrogen receptors. On histology, there is a classical association with low-grade or highly differentiated lesions as highly differentiated invasive carcinoma, ADH/DCIS, and to the other B3 lesions as classical LN. There are often associated calcifications and, therefore FEA is sometimes the only biopsy target at mammography.

#### Biology of FEA

FEA seems to be associated with a very slight increased breast cancer risk (1–2 times). Underestimation of risk is associated with ADH at MIBB.

Lesions found after FEA on breast core-needle CNB and VAB are mainly ADH and low-grade DCIS, while invasive carcinoma (in most instances highly differentiated) can occur but less frequent. Recommendation of current guidelines is increasingly in favor of surveillance if the lesion is small and the radiological findings were completely removed by CNB or VAB. Table 4 summarizes the literature update on FEA since 2015.

**Table 4:** Summary of the recent literature on FEA since 2015

Author and year	Number of patients analyzed or type of publication if no patients have been analyzed (e.g., review or comment)	Findings	Conclusions
Acott and Mancino 2016 [23]	*n* = 46 Isolated FEA on CNB	Underestimation FEA on CNB 2%	May warrant close surveillance
Berry et al. 2016 [24]	*n* = 27 FEA on CNBs	Underestimation FEA on CNB 11%	Only patients with a history of breast cancer or pure, prominent FEA on CNB disease should proceed to excisional biopsy
Chan et al. 2018 [25]	*n* = 195 Isolated FEA on CNB		Non-operative management of biopsy-proven FEA can be considered in the absence of ADH and radiology–pathology discordance
Dialani et al. 2014 [26]	*n* = 37 Isolated FEA on VAB	Upgrade FEA on VAB (6.9%)	If there are no residual microcalcifications following CNB, imaging follow-up as an alternative to surgery may be a reasonable option
Lamb et al. 2017 [27]	Pure FEA on CNB/VAB Upgrade (*n* = 200) and follow-up (*n* = 8)	Upgrade FEA in CNB/VAB (2.5%)	Recommend surveillance rather than surgical excision
McCroskey et al. 2018 [28]	FEA on VAB (43) FEA/ADH on VAB (18) FEA/LN on VAB (8)	No upgrade	Excision may not be necessary for pure FEA and FEA with atypical ductal hyperplasia limited to ≤ 2 terminal duct-lobular units, if at least 90% of calcifications have been removed on biopsy
Rudin et al. 2017 [29]	Metanalysis of 32 studies	Management change in 25%	Recommendation of OE after FEA on CNB
Samples et al. 2017 [30]	Interobserver diagnostic variability	Wide variation in the diagnosis of FEA	Diagnostic criteria may vary
Schiaffino et al. 2018 [31]	Upgrade FEA on VAB (*n* = 48)	Upgrade FEA in VAB (2%)	Surgical excision may not be necessary in patients with VAB diagnosis of isolated FEA, without residual microcalcifications post-procedure and with concordant mammography
Yamashita et al. 2016 [32]	Interobserver diagnostic variability	Morphological criteria as nuclear ellipticity for columnar cell lesion	Consequent diagnostic criteria
Yu et al. 2015 [22]	*n* = 128 Upgrade FEA on VAB	No upgrade	No OE is necessary if calcification is removed

### Voting

If a CNB returned FEA on histology,

65% of the participants thought the lesion should be excised. 75% thought therapeutic VAB excision was acceptable and 22% thought therapeutic open surgical excision should be performed.

If a VAB returned FEA on histology,

3% of the participants thought that therapeutic open surgical excision should be performed and 97% thought that surveillance was adequate (Table 9).

### Consensus recommendation of the panel

A lesion containing FEA which is visible on imaging should undergo excision with VAB. Thereafter surveillance is justified (Table 10).

### Classical lobular neoplasia

#### Histological criteria

Lobular neoplasia (LN) includes a large spectrum and continuum of atypical intralobular proliferations of the TDLs of the breast, consisting of non-cohesive proliferating cells. Under the term “Classical Lobular Neoplasia,” the consensus conference discussed the two lesions defined by the WHO classification as classical lobular carcinoma in situ (LCIS) and atypical lobular hyperplasia (ALH), both of which represent the large majority of lobular neoplasia. ALH/LCIS are characterized by non-cohesive proliferations of atypical type A and/or B epithelial cells with mild-to-moderate nuclear atypia in about 85% of cases [33]. In case of LCIS, these cells expand more than 50% of the acini in a terminal duct-lobular unit (TDLU), while in ALH this affects less than 50%. When diagnosed on minimal invasive biopsy (VAB), these lesions are reported as B3 by the pathologist. In case of diagnostic difficulty in the histological diagnosis, the use of a combined immunohistochemistry with E-Cadherin and Catenin p120 is useful to rule out morphological differential diagnoses especially as solid DCIS.

In contrast, the rare morphologic variants including pleomorphic LN which demonstrates marked nuclear pleomorphism equivalent to that of high-grade ductal carcinoma in situ (DCIS), with or without apocrine features. A florid LN along with marked distention of TDLUs or ducts, often with accompanying mass formation and comedo type necrosis, are reported as B5a as DCIS and are not discussed as LN in this consensus report. The underlying rationale is that in contrast to LCIS and ALH, 25–60% of cases with LN (B5a category) variants on CNB/VAB are found to upgrade to carcinoma on excision [34–36]. The reproducibility of all LIN ALH versus LCIS is poor, the prognostic significance between LIN1,2 is not supported by evidence, so it is not endorsed by current European guidelines (AGO [37]). It is a simplified and practical way to categorize these lesions as B3 (e.g., as classical LN) and B5a (as pleomorphic or florid LN) especially on CNB and VAB.

### Biological behavior

ALH/LCIS has to be considered as both, a risk factor and a non-obligate precursor of invasive breast carcinoma conferring an 8 to 10 times relative risk compared to the general population [38, 39]. The absolute risk of either lobular or ductal breast cancer is in the range of 1–2% per year with a cumulative long-term rate of more than 20% at 15 years and 35% at 35 years [39, 40]. The risk is bilateral with ipsilateral predominance [41, 42].

Until now, no single histopathological or clinical factor alone has been identified which could link the development of breast cancer to a histological diagnosis of classical LN.

### Risk of breast cancer at CNB/VAB

The management of patients with classic LN when diagnosed on MIBB (CNB/VAB) has been controversial due to a wide range (0–60%) of reported upgrade rates to DCIS or invasive carcinoma on excision. Those rates result above all from disregarding radiological–pathological correlation [43–46]. LCIS and ALH are infrequently seen as the sole finding in CNB or VAB accounting for 0.5–2.9% of biopsies taken for histologic assessment of mammography-detected lesions. Therefore, recent studies of classic LCIS and ALH as incidental finding in cases where a different benign pathological lesion in the same biopsy has been proved to represent the correlation to the radiological biopsy target with concordant imaging findings report very low (~ 1–4%) excisional upgrade rates of classic LCIS and ALH to carcinoma. Regarding ALH, the largest study showed a relative risk of 8.0 for women with 3 or more foci of ALH compared to 3 or 5 for women with 1 or 2 foci, respectively. The upgrade rates for classical LCIS are generally higher (13% to 18%) when LCIS represented the radiologic target as calcification and still higher for mass lesions and calcification on imaging with radio-pathological discordance [47–49]. Current (AGO [37]) guidelines in favor of surgical management of classical LN include the presence of another B3 lesion, another lesion indicative for excision alone, the presence of a visible or mass lesion or any discordant lesions between histology and imaging (AGO [37]). Table 5 summarizes the literature update on classical LN in CNB/VAB since 2015.

**Table 5:** Summary of the recent literature on LN since 2015

Author and year	Number of patients analyzed or type of publication if no patients have been analyzed (e.g., review or comment)	Findings	Conclusions
Calhoun et al. 2016 [50]	*n* = 76 on CNB Upgrade after 15 years follow-up	10 cases (13%) with upgrade	The extent of LN in CNB may be an indicator of the likelihood of upgrade to carcinoma
Donaldson et al. 2018 [15]	*n* = 393 on CNB with ADH/LN Upgrade rate and follow-up (87 months)	Upgrade in *n* = 181 (46%) The 7-year cumulative breast cancer incidence was 9.9%	Multiple foci do not influence BC development Close clinical and radiologic follow-up for more than 5 years in this patient population
Fives et al. 2016 [51]	*n* = 25 LN on CNB accompanying fibroadenomas	Upgrade in 1 case (5%)	Rare upgrade
King et al. 2015 [40]	*n* = 1004 with /wo chemoprevention Median follow-up 81 months	10-Year cumulative risk 7% With chemoprevention 21% (3.2% per year) with no chemoprevention	Chemoprevention reduced BC risk Volume of disease, (ratio of slides with LCIS to total number of slides) was associated with breast cancer development (*p* = 0.008)
Mao et al. 2017 [52]	BC risk in LN -Hormone receptor status -Skin color		LN was higher in HR positive and in black patients
Maxwell et al. 2016 [53]	*n* = 392 pure LN 326 with OE	Upgrade to pleomorphic LN In 23/326 cases (7%)	Screen detected LN -In younger women -Unilateral -Non-pleomorphic
Nakhlis et al. 2016 [54]	*n* = 77 on CNB	Upgrade in 2 of 77 cases (2%)	Routine excision is not indicated for patients with pure LN on CB and concordant imaging findings
Renshaw and Gould, 2016 [4]	*n* = 69 CNB with LN Upgrade Follow-up	Upgrade in 17 of 69 cases (25.8%)	Immediate BC risk is higher for ADH than LN Long-term BC risk is higher for LN than ADH
Schmidt et al. 2018 [55]	*n* = 178 on CNB 115 OE 54 Surveillance (55 months follow-up)	Upgrade in 13/115 cases (11%) 1/54 Cases developed BC after follow-up (2%)	Low-upgrade rate and low BC risk
Sen et al. 2016 [56]	*n* = 447 (ALH and LCIS)	Upgrade ALH 2.4% Upgrade LCIS 8.4%	Excision is recommended for LCIS on CNB and for ALH surveillance at 6, 12, and 24 months
Susnik et al. 2016 [47]	*n* = 302 of 370 Upgrade after OE	Upgrade In 3.5% (8/228) pure LN lesions In 26.7% in “LCIS variants” (4/15) in 28.3% in LN with ductal atypia (15/53)	LN with non-classic morphology or with associated ductal atypia requires surgical excision, this can be avoided in pure LN
Xie et al. 2017 [57]	Survival outcome in SEER database (*n* = 208 + 5756 cases) Bilateral or partial mastectomy	OS after partial mastectomy without radiotherapy was not inferior to patients who underwent bilateral prophylactic mastectomy	Low breast cancer-specific mortality in patients with LCIS, therefore aggressive prophylactic surgery like bilateral prophylactic mastectomy should not be advocated for most patients with LCIS

### Voting

If a CNB returned Classical LN on histology,

69% of the participants thought the lesion should be excised. 50% thought therapeutic VAB excision was acceptable and 41% thought therapeutic open surgical excision should be performed.

If a VAB returned Classical LN on histology,

12% of the participants thought that therapeutic open surgical excision should be performed and 84% thought that surveillance was adequate (Table 9).

### Consensus recommendation of the panel

A lesion containing classical LN, which is visible on imaging should undergo excision with VAB. Thereafter surveillance is justified if there is no pathological–radiological discordance and no residual lesion.

In contrast, morphologic variants of LN (LIN 3, pleomorphic LCIS, and florid LCIS), which are reported as B5a lesions should undergo OE (Table 10).

### Papillary lesions

#### Histology and clinical presentation of PL

On imaging, intraductal papillomas vary in size and in presentation showing a spectrum of mass lesions to cystic and calcified lesions. Histology demonstrates a papillary proliferation as the basis with a central fibrovascular core containing ductal and myoepithelial cells. In case of any histological uncertainty regarding the presence of myoepithelial cells, the use of immunohistochemistry (p 63, basal cytokeratins, and estrogen receptors) is helpful. In the current WHO classification of breast tumors, papillary lesions are divided into (a) papillomas, (b) papillomas with atypia (ADH or classical LN), both belonging to the B3 category at MIBB (small solitary papillomas (< 2 mm) can be categorized as B2 lesion, if the lesion is completely surrounded by a duct structure) and to (c) papillomas with DCIS or papillomas completely involved by more extended DCIS (encapsulated papillary carcinoma), and finally (d) solid papillary carcinoma belonging to B4 or B5a category. Table 6 summarizes the literature update on B3 papillary lesions since 2015.

**Table 6:** Summary of the recent literature on PL since 2015

Author and year	Number of patients analyzed or type of publication if no patients have been analyzed (e.g., review or comment)	Findings	Conclusions
Ahn et al. 2018 [58]	*n* = 520 PL in CNB 250 with OE Upgrade	Upgrade in 17 of 250 cases (6.8%)	Factors in upgrade -Bloody nipple charge -Size on imaging ≥ 15 mm -BI-RADS≥ 4b -Peripheral location -Palpability
Armes et al. 2017 [59]	*n* = 103 PL on CNB Upgrade	Upgrade Overall in 30% With atypia in 72% Without atypia in 7%	Conservative management for those without atypia, including those without atypia in which the papillary lesion was found incidental to microcalcification in an adjacent benign lesion
Bianchi et al. 2015 [60]	Upgrade in PL lesions 46 Cases with atypia 68 Cases without atypia	Upgrade in 47.8% (22/46) cases with atypia 13.2% (9/68) without atypia	Underestimation rate in PL without atypia is lower
Khan et al. 2017 [61]	*n* = 259 PL on CNB Upgrade in OE (*n* = 147)	Upgrade 7% without atypia (8/107) 33% with atypia (13/40)	Higher upgrade in PL with atypia
Kim et al. 2016 [62]	*n* = 230 PL in CNB Upgrade In VAB (*n* = 86) In OE (*n* = 144)	Upgrade in 2.6% (6/230)	Upgrade in BI-RADS 3-4a :1.4% resp. 1.8% BI-RADS 4b-5: 13% resp. 50% No association with age and size lesion
Ko et al. 2017 [63]	*n* = 346 PL in CNB Upgrade In VAB (*n* = 211) In OE (*n* = 135)	Upgrade Overall in 2.3% If size < 1cm: 0.9%	Size of PL correlates with upgrade Close follow-up with ultrasound instead of excision
Moon et al. 2016 [64]	*n* = 65 PL in CNB Upgrade In VAB (*n* = 12) In OE (*n* = 53)	Upgrade In OE in 9% (5/53) In VAB 8% (1/12)	No recommendation
Niinikoski et al. 2018 [65]	*n* = 80 PL in CNB		Small PL in selected patients-OE can be avoided
Pareja et al.. 2016 [66]	Upgrade in OE (*n* = 171) after PL Without atypia In CNB	Upgrade In OE 2.3% (4/171)	Regardless of size, observation is appropriate at radiologic–pathologic concordant CNB
Seely et al. 2017 [67]	*n* = 107 PL in OE Upgrade after VAB (*n* = 60) CNB (*n* = 47)	Upgrade in OE After VAB in 1.6% (1/60) After CNB in 8.5% (4/47)	Higher upgrade in OE if PL is diagnosed on CNB
Tatarian et al. 2016 [68]	*n* = 16 PL in CNB Upgrade in OE	Upgrade in OE In 2/16 cases (12.5%)	Surgical excision should be considered in patients with benign papillomas
Tran et al. 2017 [69]	*n* = 43 PL in CNB Upgrade in OE	Upgrade in OE In 1/43 cases (2%)	Low-upgrade rate in OE
Wyss et al. 2014 [70]	*n* = 156 PL in CNB Upgrade In VAB (*n* = 135) and Follow-up (*n* = 21) (Median 3.5 years)	Upgrade after follow-up 1.2% (2/156)	VAB is recommended as the method of choice for removal of PL
Yamaguchi et al. 2015 [71]	*n* = 142 PL Follow-up imaging After VAB (*n* = 125) After CNB (*n* = 17)	Upgrade in OE (*n* = 17) 4/17	Discordant lesions should undergo OE
Yang et al. 2018 [72]	*n* = 116 PL (On CNB or VAB) 10 mm or smaller OE *n* = 74 Surveillance *n* = 42	Overall upgrade 11% (13/116) Upgrade after VAB (0%) Upgrade after CNB (16.5%)	Higher upgrade in OE -After CNB -Older age -Pl with atypia

### Voting

If a CNB returned PL on histology,

76.5% of the participants thought the lesion should be excised. 71% thought therapeutic VAB excision was acceptable and 23% thought therapeutic open surgical excision should be performed.

If a VAB returned PL on histology,

none of the participants (1 abstained) thought that therapeutic open surgical excision should be performed and 98% thought that surveillance was adequate (Table 9).

### Consensus recommendation of the panel

A PL lesion, which is visible on imaging should undergo excision with VAB. Larger lesions which cannot be completely removed by VAB need open excision. Thereafter surveillance is justified (Table 10).

### Phyllodes tumors (PT)

#### Histological criteria and biological behavior of PT

PTs are rare and consist of around 1–2‰ of all breast biopsies. PTs are biphasic fibroepithelial tumors varying from benign to borderline and malignant diagnostic variants. The latest WHO classification of breast tumors allows three categories depending on the number of stromal mitoses, stromal atypia, and stromal overgrowth. In some cases, the distinction between a benign cellular fibroadenoma and a benign phyllodes tumors remains despite histological diagnostic criteria problematic. Therefore, the WHO classification recommends the diagnosis of a benign fibroepithelial tumor (also categorized as B3 category) in unclear cases. Benign and borderline phyllodes tumors are B3 lesions, a malignant PT is a B5b lesion. B3 forms, particularly the benign forms of PT, are the most common, only up to 20% of all PT tumors are borderline or malignant. Risk for local recurrence at benign PT is around 10–20% and reaches up to 30% at the borderline or malignant forms. Metastatic potential depends on the form, being the highest (15–20%) at the malignant forms. Table 7 summarizes the literature update on B3 phyllodes tumors since 2015.

**Table 7:** Summary of the recent literature on PT since 2015

Author and year	Number of patients analyzed or type of publication if no patients have been analyzed (e.g., review or comment)	Findings	Conclusions
Co et al. 2017 [73]	*n* = 465 PT 281 (59.9%)benign 124 (26.4%) Borderline 64 (13.6%) malignant 384 (82%) Breast-conserving surgery (BCS) 84 (18%) Patients with mastectomy Median follow-up 85 months	Risk factors for local recurrence (1) Positive margins (*p* < 0.001) (2) BCS (*p* < 0.001) Risk factors for local metastases (1) Large tumor size (*p* = 0.008) (2) Malignant component (*p* < 0.001) Disease-free survival 99.6% (benign) 100% (borderline) 90.6% (malignant)	
Kim et al. 2017 [73]	*n* = 146 PT (benign) Surgery (*n* = 126) US-VAB (*n* = 20)	Three cases (2.1%, 3/146) had recurrence and all were in the surgery group (2.4%, 3/126)	Clinical follow-up rather than further surgery at benign phyllodes tumor diagnosed at US-VAE, if there is no residual lesion at US
Ouyang et al. 2016 [74]	*n* = 225 benign PT Surgery (*n* = 117) VAB (*n* = 108)	5-year cumulative RFS 81.6 (VAB) 8.7% (surgery) (*p* = 0.11)	No difference in DFS between OE and VAB removal
Sevinc et al. 2018 [75]	*n* = 122 PT (benign and borderline) All underwent surgical excision	No local recurrence occurred in any group Positive surgical margins in 43 (35%) Margins ≥ 10 mm in 16 patients (13%) Margins 2–10 mm in 48 patients (40%) Margins ≤ 1 mm in 15 patients (12%)	Positive resection margins did not influence local recurrence
Shaaban and Barthelmes 2017 [76]	*n* = 1702 PT Literature review (12 studies) Margin assessment 1 mm distance 10 mm distance Focal margin involvement	No difference in recurrence rates between a 1- and a 10-mm margin	The recurrence rate increases if there is focal margin involvement. 1 mm is acceptable for benign PT
Youk et al. 2015 [77]	*n* = 41 PT (benign) OE after VAB (*n* = 27) 2 Years follow-up with US (*n* = 14)	Upgrade 2/23 (8.7%) to malignant PT Residual tumor 15/27 (55%)(at VAB site) 0/14 (0%)(US follow-up)	PTs diagnosed after US-VAB should be surgically excised
Zhou et al. 2016 [78]	Sensitivity of definitive PT category in CNB versus OE	The sensitivity of CNB 4.9% (2/41) benign 4.2% (3/71) borderline 25.0% (4/16) malignant	CNB in PT category has low sensitivity

### Voting

If a CNB returned PT on histology,

98% of the participants thought the lesion should be excised. 22% thought therapeutic VAB excision was acceptable and 72% thought therapeutic open surgical excision should be performed.

If a VAB returned PT on histology,

8% of the participants thought that therapeutic open surgical excision should be performed and 88% thought that surveillance was adequate (Table 9).

### Consensus recommendation of the panel

A PT lesion, which is found by CNB, should undergo open surgical excision with clear margins. If accidentally found by VAB without any corresponding imaging finding, surveillance of a benign PT is justified, while borderline and malignant PTs require re-excision to obtain clear margins (Table 10).

### Radial scar

#### Histological features of RS

Two papers published almost at the same time described the same lesion naming that was named radial scar by Hamperl [79] and scleroelastotic lesion by Eusebi et al. [80]. More recently, the definition of complex sclerosing lesion (CSL) has been proposed. RS is characterized by a central area mimicking a scar, containing one to several ducts showing obliterative mastopathy, and surrounded by elastic fibers. In addition, other ducts converge into the scar-like area in a stellate fashion. The epithelium lining the latter ducts may show a great variety of changes, the most frequent being benign epitheliosis (usual ductal hyperplasia). The central scar-like area together with stellate appearance of the outer ducts easily mimics an invasive carcinoma, both on radiological and histological grounds. RS can be detected during screening mammography and now even more often by tomosynthesis, therefore sampled by CNB or by VAB. There is general agreement that RS alone is a benign lesion, but RS can be occasionally associated with carcinoma. When RS is associated to atypia (such as flat epithelial atypia (FEA), atypical ductal (ADH), or lobular neoplasia (classical LN)), management can the same as recommended in cases of atypia alone. Management is more controversial in cases without atypical lesions. In these cases, upgrade of cancers is associated with architectural distortions and larger masses (≥ 10 mm), calcifications, and older age [69, 71]. The recently published data suggest that in cases of RS diagnosed using CNB or VAB, it must be taken into consideration that (a) accurate and detailed radiological–pathological correlations must be obtained; (b) lesions < 10 mm have lower rate of cancer upgrading; (c) histology is vital in the evaluation of presence or absence of atypical features within the lesion. Table 8 summarizes the literature update on radial scar since 2015.

**Table 8:** Summary of the recent literature on RS since 2015

Author and year	Number of patients analyzed or type of publication if no patients have been analyzed (e.g., review or comment)	Findings	Conclusions
Donaldson et al. 2016 [81]	*n* = 37 RS upgrade in OE	Upgrade in OE 31/37 (84%, benign) 2/37 (5%, ADH) 3/37 (8%, LN classic) 1/37 (3%, FEA)	Low upgrade in OE at isolated radial scar on preoperative CNB/VAB
Ferreira et al. 2017 [82]	*n* = 113 RS 25 (CNB) 88 (VAB)	Upgrade in OE 22/113 (20%) Risk for upgrade -Type of biopsy (CNB or VAB) -Presence of atypia -Presence of calcifications -Nr. of biopsy fragments	At VAB, the risk of upgrade and malignancy is significantly decreased and so the indication for excisional biopsy seems not to be so imperative
Hou et al. 2016 [83]	*n* = 113 RS *n* = 81 without atypia *n* = 32 with atypia	Upgrade in OE No upgrade in RS without atypia	RS without atypia on VAB has a very low risk for upgrade
Kalife et al. 2016 [84]	*n* = 100 RS on CNB 41 cases had OE	Upgrade in OE 4/41 (10%) cases with atypia No cases to malignancy	Close imaging follow-up is adequate for patients with RS/RSL without associated atypia malignancy on CNB
Kim et al. 2016 [85]	*n* = 88 RS on CNB/VAB 63 (72%) had OE	Upgrade in OE 1/63 (1.5%)	Isolated radial scar may not warrant routine surgical excision given relatively low cancer upgrade rates
Leong et al. 2016 [86]	*n* = 219 RS on CNB 161 (74%) had OE	Upgrade in OE 1/161 (0.6%)	Surgical excision is unnecessary if radial scar is found at CNB without an associated proliferative lesion but is still indicated when radial scar is associated with atypical ductal hyperplasia or lobular neoplasia
Li et al. 2016 [87]	*n* = 403 pure RS on CNB 220 (54.6%) had OE	Upgrade in OE 2/220 (0.9%) malignancy 44/220 (20%) ADH 13/220 (5.9%) classical LN	Conservative follow-up with imaging rather than surgical excisions may be more appropriate for isolated RS
Miller et al. 2014 [88]	*n* = 131 pure RS on CNB All had OE	Upgrade in OE 2 /131 (1.5%) malignancy 22/131 (17%) high-risk B3 lesion	Excision of RS to rule out associated invasive carcinoma is not warranted, given a 1% rate of upgrade at excision
Nassar et al. 2015 [89]	*n* = 38 RS Upgrade in OE	Upgrade in OE 4/38 (10%) malignancy 7/38 (18%) high-risk lesions (1xADH, 6xclassical LN)	Open excision for RS larger than 1.0 cm with worrisome radiographic findings or with radiologic and pathologic discordance is recommended
Park et al. 2016 [90]	*n* = 10 pure RS on CNB Upgrade in OE	No upgrade in OE	Pure RS on CNB may not need OE

### Voting

If a CNB returned RS/CSL on histology,

85% of the participants thought the lesion should be excised. 72% thought therapeutic VAB excision was acceptable and 26% thought therapeutic open surgical excision should be performed.

If a VAB returned RS/CSL on histology,

2% of the participants thought that therapeutic open surgical excision should be performed and 98% thought that surveillance was adequate (Table 9).

**Table 9:** Summary of the voting for each pure B3 lesion

	A diagnosis of a visible (on imaging by mammography or ultrasound) lesion by means of spring-loaded core biopsy (14–18 g) has been made			What method of excision should be chosen			A lesion has been removed by means of VAB and the lesion on imaging seems to be removed			
	The lesion should be removed	The lesion should not be removed	Undecided/abstain	VAB is acceptable	Open biopsy should be preferred	Undecided/abstain	An open re-excision should be performed	A repeat VAB should be performed	Wait and see is justified	Undecided/abstain
ADH	35 (100%)	0	0	8 (21.1%)	28 (73.7%)	2 (5.3%)	20 (51.3%)	0	18 (46.2%)	1 (2.6%)
FEA	43 (65.2%)	14 (21.2%)	9 (13.6%)	51 (75%)	15 (22.1%)	2 (2.9%)	2 (2.9%)	0	67 (97.1%)	0
LN	46 (68.7%)	9 (13.4%)	12 (17.9%)	34 (50%)	28 (41.2%)	6 (8.8%)	8 (11.6%)	0	58 (84.1%)	3 (4.3%)
PL	39 (76.5%)	9 (17.6%)	3 (5.9%)	37 (71.2%)	12 (23.1%)	3 (5.8%)	0	0	52 (98.1%)	1 (1.9%)
PT	48 (98%)	1 (2%)	0	11 (22%)	36 (72%)	3 (6%)	4 (7.8%)	0	45 (88.2%)	2 (3.9%)
RS	28 (59.6%)	15 (31.9%)	4 (8.5%)	37 (80.4%)	7 (15.2%)	2 (4.3%)	2 (4.3%)	0	42 (89.4%)	3 (6.4%)

### Consensus recommendation of the panel

A RS/CSL lesion, which is visible on imaging should undergo therapeutic excision with VAB. Thereafter surveillance is justified (Table 10).

**Table 10:** Summary of the recommendations for each B3 lesion

	Diagnosis made by CNB	Diagnosis made by VAB
ADH	OE	OE. surveillance can be considered in a few special situations after discussion at the MDM
FEA	VAB to complete removal of the lesion visible in any imaging method	Surveillance is justified if the radiological lesion has been removed
LN	OE or VAB (remove US-visible lesion)	OE or high-risk surveillance if the radiological lesion has been removed
PL	Remove by VAB	
PT	OE. Free margins in borderline and malignant PTs	Follow-up in completely excised benign PTs surveillance is justified
RS	VAB or OE of visible lesion	Surveillance is justified if the radiological lesion has been removed

*VAB* usually the lesion should not exceed 2.5 cm in diameter. For larger lesions, OE is preferred, *LN* only classical type. LN pleomorphic, LIN 3, LN extended, and LN with necrosis are defined as B5a lesions and should undergo OE, *PL* with atypia: Such a lesion should not be classified as papilloma, but rather as FEA or ADH according to the type of atypia found

Tables 9 and 10 show the summaries of the votings and the recommendations for each B3 lesion.

## Discussion

The panel agreed that underestimation rates should be below 5% for IC and below 10% for DCIS. If a certain B3 lesion shows an upgrade rate of more than 10%, in general surveillance was not recommended. Computer-aided decision making would be of interest. Bahl et al. [91] show the potential of machine learning methodology in the field of high-risk breast lesions predicting the risk of upgrade (editorial by Shaffer [92]).

Other recommendations [93, 94] favor recommendations from the consensus meetings. They do not explicitly propose VAB as we do, probably due to the fact, that VAB is not so well established in other countries yet.

The 2018 recommendations confirm largely the 2016 recommendations. Results presented in the recent literature confirm the 2016 recommendations for surveillance after a B3 lesion diagnosed by VAB or CNB, especially for FEA, RS, PL, and PT. Upgrade rates are high in ADH and in LN which are not only focal or an incidental finding especially if pathological–radiological concordance is not given. LN lesions with pleomorphic, extended features, and LN with necrosis should be reported as B5a lesions and should undergo OE as DCIS. Our recommendations for ADH are slightly less liberal in 2018 than in 2016 and tend more towards OE.
