# Correction to: Second International Consensus Conference on lesions of uncertain malignant potential in the breast (B3 lesions)

**DOI:** 10.1007/s10549-019-05287-9

**Published:** 2019-05-31

**Authors:** Christoph J. Rageth, Elizabeth A. M. O’Flynn, Katja Pinker, Rahel A. Kubik-Huch, Alexander Mundinger, Thomas Decker, Christoph Tausch, Florian Dammann, Pascal A. Baltzer, Eva Maria Fallenberg, Maria P. Foschini, Sophie Dellas, Michael Knauer, Caroline Malhaire, Martin Sonnenschein, Andreas Boos, Elisabeth Morris, Zsuzsanna Varga

**Affiliations:** 1 Département de Gynécologie et d’Obstétrique, Centre du sein, Hôpitaux Universitaires de Genève, Bd de la Cluse 30, 1211 Geneva 14, Switzerland; 2 The Rose Centre, St George’s University Hospitals NHS Foundation Trust, Perimeter Road, London, SW17 0QT UK; 3 Breast Imaging Service, Department of Radiology, Memorial Sloan Kettering Cancer Center, 300 E 66th St, New York, NY 10065 USA; 4 Department of Medical Services, Institute of Radiology, Kantonsspital Baden, im Ergel, 5404 Baden, Switzerland; 5 Zentrum Radiologie der Niels-Stensen-Kliniken; Marienhospital Osnabrück, Bischofsstraße 1, 49074 Osnabrück, Germany; 6 Institut für Pathologie am Dietrich-Bonhoeffer-Klinikum, Salvador-Allende-Straße 30, 17036 Neubrandenburg, Germany; 7 Brust-Zentrum Zürich, Seefeldstr. 214, 8008 Zurich, Switzerland; 8 Interventional and Pediatric Radiology, Department of Diagnostic, Inselspital, University Hospital Bern, Freiburgstrasse 10, 3010 Bern, Switzerland; 9 Department of Biomedical Imaging and Image-guided Therapy, Allgemeines Krankenhaus, Medical University of Vienna, Währinger Gürtel 18-20, 1090 Vienna, Austria; 10 Department of Radiology, University Hospital, Ludwig Maximilian University Munich, Marchioninistr. 15, 81377 Munich, Germany; 11 Department of Biomedical and Neuromotor Sciences, Unit of Anatomic Pathology at Bellaria Hospital, University of Bologna, Via Altura 3, 40139 Bologna, Italy; 12 Clinic of Radiology and Nuclear Medicine, University Hospital Basel, University of Basel, Petersgraben 4, 4031 Basel, Switzerland; 13 Breast Center St. Gallen, Cantonal Hospital St. Gallen, Rorschacher Str. 95, 9007 St. Gallen, Switzerland; 14 Imaging Department, Institut Curie, PSL Research University, Paris, France; 15 Division of Radiology, Breast Center Bern (Brustzentrum Bern), Klinik Engeried, Lindenhofgruppe AG, Riedweg 15, 3012 Bern, Switzerland; 16 Institute of Diagnostic and Interventional Radiology, University Hospital Zurich, Rämistr. 100, 8091 Zurich, Switzerland; 17 Institute of Pathology and Molecular Pathology, University Hospital Zurich, Switzerland Schmelzbergstrasse 12., 8091 Zurich, Switzerland; 18 Ringlikerstrasse 53, 8142 Uitikon Waldegg, Switzerland

## Correction to: Breast Cancer Research and Treatment (2019) 174:279–296 10.1007/s10549-018-05071-1

The article Second International Consensus Conference on lesions of uncertain malignant potential in the breast (B3 lesions), written by Christoph J Rageth, Elizabeth AM O’Flynn, Katja Pinker, Rahel A Kubik-Huch, Alexander Mundinger, Thomas Decker, Christoph Tausch, Florian Dammann, Pascal A. Baltzer, Eva Maria Fallenberg, Maria P Foschini, Sophie Dellas, Michael Knauer, Caroline Malhaire, Martin Sonnenschein, Andreas Boos, Elisabeth Morris, Zsuzsanna Varga, was originally published electronically on the publisher’s internet portal (currently SpringerLink) on November 30, 2018 without open access.

With the author(s)’ decision to opt for Open Choice the copyright of the article changed on May 30, 2019 to © The Author(s) 2018 and the article is forthwith distributed under the terms of the Creative Commons Attribution 4.0 International License (http://creativecommons.org/licenses/by/4.0/), which permits use, duplication, adaptation, distribution and reproduction in any medium or format, as long as you give appropriate credit to the original author(s) and the source, provide a link to the Creative Commons license and indicate if changes were made.

The original article has been corrected.

